# Predicting Adaptive Phenotypes From Multilocus Genotypes in Sitka Spruce (*Picea sitchensis*) Using Random Forest

**DOI:** 10.1534/g3.112.002733

**Published:** 2012-09-01

**Authors:** Jason A. Holliday, Tongli Wang, Sally Aitken

**Affiliations:** * Department of Forest Resources and Environmental Conservation, Virginia Polytechnic Institute and State University, Blacksburg, Virginia 24061, and; † Department of Forest Sciences, University of British Columbia, Vancouver, BC, Canada, V6T 1Z4

**Keywords:** Random Forest, adaptation, association mapping, epistasis, phenology, cold hardiness, GenPred, shared data resources

## Abstract

Climate is the primary driver of the distribution of tree species worldwide, and the potential for adaptive evolution will be an important factor determining the response of forests to anthropogenic climate change. Although association mapping has the potential to improve our understanding of the genomic underpinnings of climatically relevant traits, the utility of adaptive polymorphisms uncovered by such studies would be greatly enhanced by the development of integrated models that account for the phenotypic effects of multiple single-nucleotide polymorphisms (SNPs) and their interactions simultaneously. We previously reported the results of association mapping in the widespread conifer Sitka spruce (*Picea sitchensis*). In the current study we used the recursive partitioning algorithm ‘Random Forest’ to identify optimized combinations of SNPs to predict adaptive phenotypes. After adjusting for population structure, we were able to explain 37% and 30% of the phenotypic variation, respectively, in two locally adaptive traits—autumn budset timing and cold hardiness. For each trait, the leading five SNPs captured much of the phenotypic variation. To determine the role of epistasis in shaping these phenotypes, we also used a novel approach to quantify the strength and direction of pairwise interactions between SNPs and found such interactions to be common. Our results demonstrate the power of Random Forest to identify subsets of markers that are most important to climatic adaptation, and suggest that interactions among these loci may be widespread.

The health of forest tree populations is inextricably linked with the ability of local populations to track phenotypic optimums enforced by their respective climates. With anthropogenic climate change substantially altering adaptive landscapes, tree populations are expected to suffer maladaptation, become sources rather than sinks of carbon, and ultimately be forced to adapt, migrate, or be extirpated (Savolainen *et al.* 2007; Aitken *et al.* 2008). Paleoecological data suggest that migration rates may be insufficient to realize range shifts predicted by climate-based species distribution models (Malcolm *et al.* 2002; McLachlan *et al.* 2005; Hamann and Wang 2006), and thus, the importance of adaptive evolution in response to new climatic regimes cannot be underestimated. To determine the potential for adaptation, we must first have an understanding of the genomic underpinnings of variation in climate-related phenotypic traits.

The current method of choice for elucidating the genomic determinants of complex adaptive traits is association mapping (Neale and Savolainen 2004; Gonzalez-Martinez *et al.* 2006b). Widespread temperate and boreal tree species are well suited to this approach, which has been successfully demonstrated in a variety of species (Gonzalez-Martinez *et al.* 2007, 2008; Ingvarsson *et al.* 2008; Eckert *et al.* 2009a; Holliday *et al.* 2010; Wegrzyn *et al.* 2010). Conventional analytical approaches to association mapping studies estimate the marginal effect of individual polymorphisms on the trait of interest (Yu *et al.* 2006), but these approaches are not designed to predict phenotypes from multilocus genotypes nor to detect interactions among loci. There has been increasing interest in the use of multiple regression-like models that take all single-nucleotide polymorphisms (SNPs) as predictor variables simultaneously. This approach is most frequently applied to mapping populations generated by controlled crosses for the purposes of selecting germplasm for deployment or further crosses in breeding programs—so-called “genomic selection.” The goal of these methods is not so much to identify trait-associated SNPs as it is to saturate the genome with markers such that most quantitative trait loci will be captured through linkage disequilibrium (Meuwissen *et al.* 2001; Jannink *et al.* 2010). The extension of this approach to trees has been suggested and the first test cases recently reported (Grattapaglia and Resende 2011; Resende *et al.* 2012)

Although genomic selection has shown great promise in the fields of animal and plant breeding, it is not currently feasible as a tool for conservation genetics in natural populations because of the rapid decay of linkage disequilibrium (LD), which would necessitate hundreds of thousands to millions of markers be genotyped. Ideally, one would be able to identify a small core set of ecologically relevant markers that comprise much of the adaptive variation segregating in the wild, which could then be targeted by land managers to assess the adaptive portfolio of local populations. The decision tree algorithm ‘Random Forest’ (RF) (Breiman 2001) is an alternative method to uncover the relative importance of SNPs to the expression of adaptive traits and to predict the phenotypes of adaptive traits by accounting for both the cumulative effect of individual SNPs and the effect of all forms of interactions among SNPs without the need to define these terms in the model. The principle behind RF is to build decision trees (or regression trees in the case of continuous dependent variables) by categorizing observations using multiple predictor variables. In contrast to conventional decision and regression tree approaches, RF constructs a large number of trees (the ‘forest’) by introducing two layers of randomness—random bootstrap sampling of the data and random selection of a subset of predictors for splitting at each node—which improves predictive accuracy (Cordell 2009). The relative importance of individual SNPs is assessed by permutation: SNPs with no effect on phenotypes will not change the prediction of the tree when their values are permuted, whereas SNPs with explanatory power will. Several studies have used RF to detect loci involved in binary disease phenotypes in humans (Bureau *et al.* 2005; Goldstein *et al.* 2010; Chen *et al.* 2011;), and one study extended the RF approach to quantitative variation in resistance to powdery mildew in *Arabidopsis* (Nemri *et al.* 2010).

In addition to accounting for multilocus combined effects, RF can be used to test for interactions (*i.e.*, epistasis) among SNPs. Epistasis is a fundamental component of complex trait variation that may be an important driver of adaptation, but such effects are difficult to quantify because of the computational burden of testing all possible pairwise interactions for hundreds or thousands of loci (Phillips 2008). In the context of association mapping, several methods have been developed to detect interacting SNPs (Hahn *et al.* 2003; Lunn *et al.* 2006; Moore *et al.* 2006; Zhang and Liu 2007), but these methods are still computationally intensive (and in some cases intractable) for datasets comprising more than a few hundred SNPs (Cordell 2009). By contrast, RF offers an efficient framework to test for epistasis, which does not require that all possible pairwise interactions be specified in the model. Any predictor SNP in an RF model that has a significant interaction, even in the absence of a main effect, should be assigned high importance because the phenotype prediction will change when its values are permuted. Lunetta *et al.* (2004) simulated interacting disease risk-associated SNPs and found that selecting SNPs on the basis of their importance value from an RF analysis lead to improved detection of epistasis. Jiang *et al.* (2009) applied a sliding-window approach with RF to reduce the number SNPs tested for interactions in a case-control binary trait study involving a huge number of SNPs. However, to the best of our knowledge no report has been made that tested SNP interactions for quantitative traits using RF. In particular, no method is available to detect the directions of such interactions between each SNP pair.

We recently completed an association study in Sitka spruce encompassing 202 candidate genes related to local adaptation, and identified numerous genotype-phenotype associations (Holliday *et al.* 2010). Among 339 SNPs tested, 35 were significantly associated with either timing of budset or cold hardiness. The current paper uses the RF framework to analyze the same dataset by building an integrated model to predict phenotypes on the basis of their multilocus genotypes, to select a subset of SNPs with the greatest predictive power, and uses a novel approach to detect the extent and direction of epistasis among these predictive SNPs.

## Materials and Methods

We previously reported association mapping of climate-related traits in Sitka spruce (*Picea sitchensis*) (Holliday *et al.* 2010). In this study, the Illumina GoldenGate platform was used to genotype a range-wide mapping population of 410 individuals, which were also measured for timing of budset (defined as the Julian date on which the first bud scales were visible, assessed weekly from July 7 through December 31, 2007) and cold hardiness (measured on December 1, 2007, using electrolyte leakage as a proxy for cell death after Hannerz *et al.* (1999)). Sampled populations, their origins, and cluster assignments are described in supplementary materials (supporting information, Table S1). Substantial variation both within and among populations was observed for both of these traits. By using a mixed model approach that accounts for population structure, we found 35 SNP loci (from a panel of 339 successful SNP assays) to be associated with timing of autumn budset, autumn cold hardiness, or both.

We used the data from Holliday *et al.* (2010) to build a predictive model for adaptive traits, for which dependent variables consisted of the two phenotypes described previously, and independent variables consisted of 339 SNPs that passed quality control and were polymorphic. Candidate gene names and genotype/phenotype data can be found in supplementary materials (Table S2 and Table S3, respectively). The procedure to build the random forest is as follows: (1) take a bootstrap sample of observations (*i.e.*, a random sample with replacement; ∼64% by default) from the total population, leaving the rest ‘out of bag’ (OOB) to be used for independent predictions and error checking; (2) at each successive node randomly sample a subset of SNPs (approximately the square-root of the total by default, but tuned for optimal values), and use the SNP that minimizes predictive error to split observations at that node into ‘branches’; (3) continue this process until there is no further improvement in predictive accuracy of terminal ‘leaves’; (4) repeat the aforementioned steps to build a large number of regression trees forming the random forest; (5) predict the phenotypes for the OOB samples through the trees for which they were OOB and take the average of the predictions across trees as the final phenotype prediction for a given sample. The importance value reflecting the relative contribution of each SNP to the model is estimated by randomly permuting its values and recalculating the predictive accuracy of the model. The difference of the model accuracy before and after the random permutations, averaged over all trees in the forest, tells us how important that predictor is for determining the outcome. The amount of variance explained by the predicted values relative to the total amount of variation in the observed phenotypes provides an estimate of the explanatory power of the model.

Because population structure can increase type-one error rates (false-positives) in association studies, model predictions are more credible with population structure removed. Holliday *et al.* (2010) used *Structure* software (Pritchard *et al.* 2000) to show that three populations provides the most parsimonious solution to population subdivision across the species range in Sitka spruce. To remove the effect of population structure, we regressed the two phenotypic traits on estimates of cluster membership derived from replicate runs of *Structure* (that is, the Q matrix from *Structure*). The sums of the general mean and the residue of each observation from this regression were used as dependent variable for RF analysis. However, as Sitka spruce spans a wide latitudinal range, with latitude highly correlated with climatic variables, but is restricted to a narrow longitudinal range, population structure covaries strongly with climate gradients. The removal of the population structure may thus result in overadjustment (false-negatives) for the effect of local adaptation to climate. Therefore, phenotype prediction was conducted both with and without an adjustment for population structure.

For this analysis, 1500 regression trees were constructed in the model, although no further improvement was found after the number of trees increased to 1000. The model was initially run with all 339 SNPs as predictors (the full model), followed by runs using various numbers of selected SNPs based on their relative importance values to optimize the model. In previous studies, the importance value of each SNP assigned by the full model was used to rank the SNPs. However, as RF accounts for interactions, the ranking changes when the combination of the SNPs changes (*i.e.*, adding or dropping one or more SNPs to the model). To consider the effect of interactions among the SNPs on phenotype prediction accuracy and on the ranking of their importance, we developed a backward purging approach to purge the least important SNP step by step. The minimum number of SNPs that can explain the maximum amount of variance in observed phenotypes was around 20. We thus applied this approach starting with a highly bifurcating model with the top-ranked 50 SNPs included to avoid excluding those SNPs with large interactions but insignificant main effects. At each step, the model run was repeated three times, and the SNP with the smallest importance value was eliminated from the model. The amount of variance explained by each successive model was averaged over the three runs. This step was repeated until there were only two SNPs remaining in the model (the minimum number of predictor variables that can be used).

To validate the effectiveness of our approach in selection of most important SNPs for the adaptive traits, we compared the effect of our selected SNPs on prediction of phenotypes with randomly selected SNPs. For this, RF model was run with bootstrap samples of SNPs (*i.e.* random combinations) with sample sizes of 2, 5, 10, 15, and 20 SNPs. These were referred to as ‘random’ models. The random sampling and model runs were repeated 10 times with each sample size, from which the average percentages of the amount of variance explained were calculated for each sample size. All RF analyses were performed using the R version of RF (Liaw and Wiener 2002).

Although RF accounts for epistasis without directly testing all possible pairwise combinations of loci, which is computationally efficient, it does not identify specific interacting SNPs. We have extended the method to enable direct testing of putatively interacting SNPs by removing SNPs with high importance values one-by-one and recalculating the importance values of remaining SNPs. If two SNPs interact, the absence of one of them would increase or decrease the importance values of the remaining SNP. Therefore, we can determine the amount and the direction (negative or positive) of the two-way interactions among important SNPs through examining the changes in their importance values when a SNP is removed from the model. We did this for each of the 20 most important SNPs to quantify both synergistic epistasis (*i.e.*, when the importance of a SNP decreases upon removal of another SNP) and disruptive epistasis (*i.e.*, when the importance value of a SNP increases upon removal of another SNP). This procedure was repeated five times, and the changes in the importance values were calculated for each locus in each run. The student's *T*-tests were applied to determine whether the changes in the importance values were significant. The *P*-values for the tests were adjusted for multiple testing using the *qvalue* package in R [http://www.r-project.org/ (Storey and Tibshirani 2003]. Tests quantifying interactions among SNPs were carried out only for the dataset with population structure removed. Interactions networks were displayed using Cytoscape software (Shannon *et al.* 2003).

To determine whether interacting SNPs were also in LD, we used our unphased genotypic data to calculate the probability that genotypes at pairs of loci are independent by using the composite LD test implemented in Genepop software (Rousset 2008). *P*-values obtained from this test were adjusted for the false-discovery rate (FDR).

## Results

### Phenotype prediction

**Figure 1:**
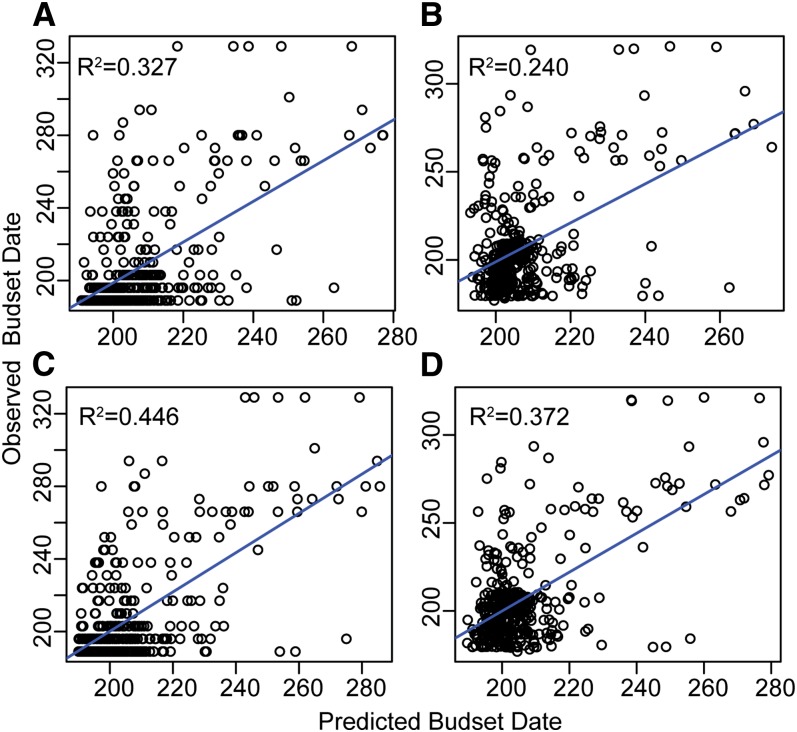
Relationship between observed budset dates (given as days from January 1) and those predicted by RF using all 339 SNPs (A) without adjusting for population structure and (B) with adjustment. The 20 SNPs with highest importance [% mean squared error (MSE)] were then used to similarly re-run RF both without adjusting for population structure (C) and with adjustment for population structure (D). All regressions were significant (*P* < 0.001).

**Figure 2:**
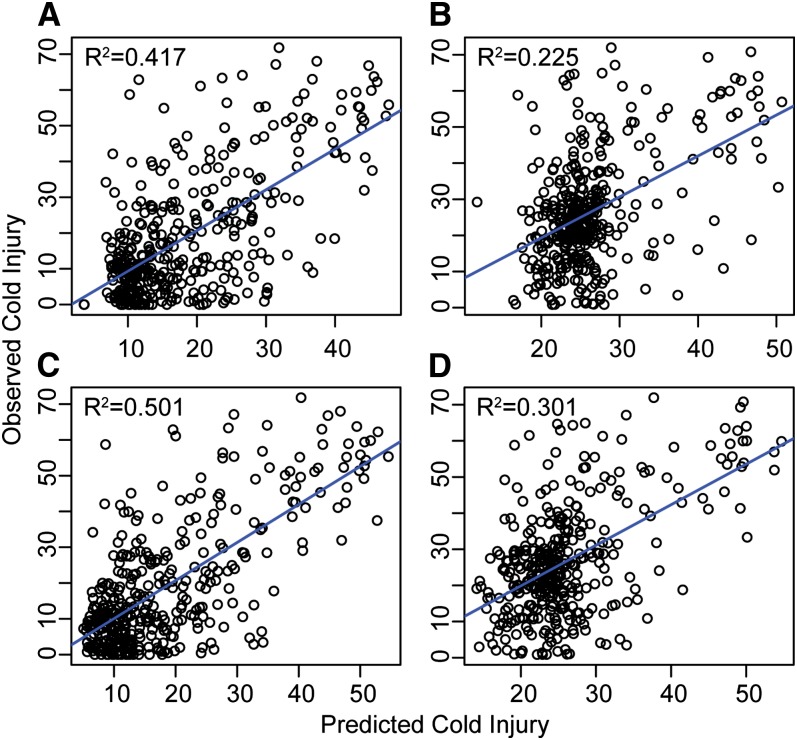
Relationship between observed cold injury (averaged across three test temperatures) and that predicted by RF using all 339 SNPs (A) without adjusting for population structure and (B) with adjustment. The 20 SNPs with highest importance (% MSE) were then used to similarly re-run RF both without adjusting for population structure (C) and with adjustment for population structure (D). All regressions were significant (*P* < 0.001).

**Figure 3:**
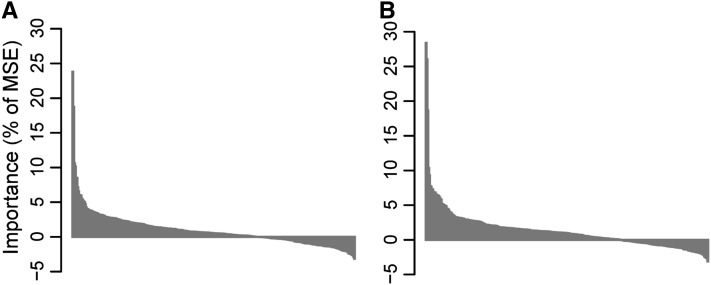
Importance values for all 339 SNPs in the study (vertical lines) for (A) budset date and (B) cold hardiness, adjusted for population structure.

When all 339 SNPs were used as predictors, RF explained 32.7% of the phenotypic variation in budset timing (Figure 1A). When budset dates were adjusted for population structure using multiple regressions before running RF, this value decreased to 24% (Figure 1B). The predictive value of the model improved when only the 20 most important SNPs were used as predictors, which were selected using the backward purging approach described in the *Materials and Methods* (Figure 1, C and D). The percentage of phenotypic variance explained (PVE) increased to 44.6% and 37.2% for the population unadjusted and adjusted models, respectively. Accounting for population structure had a more dramatic effect on prediction of cold injury, for which the unadjusted model accounted for 41.7% of variation, whereas the adjusted model accounted for only 22.5% (Figure 2, A and B). When only the top 20 SNPs were again selected, PVE was improved by 8.4% and 7.6% for the uncorrected and corrected models, respectively (Figure 2, C and 2D). These results are consistent with an increase in noise contributed by additional uninformative SNPs, which is reflected in negative importance values for a substantial fraction of the 339 SNPs for both budset (Figure 3A) and cold hardiness (Figure 3B).

**Figure 4:**
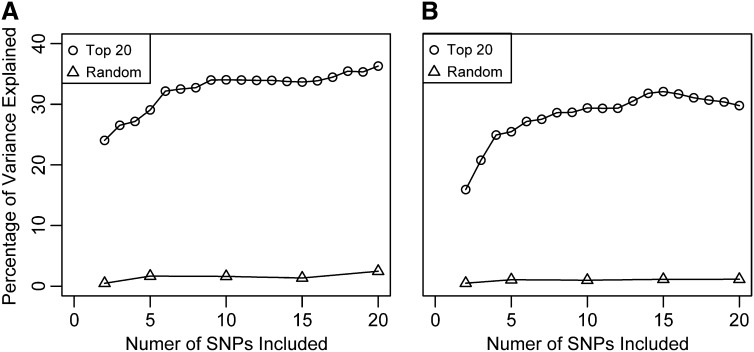
Percentages of total variance explained in (A) bud set and (B) observed cold injury with the number of SNPs included in the RF models. SNPs were selected using optimized combinations of between two and 20 SNPs from all 339 SNPs (Top 20) and using bootstrapped random samples (Random).

The result of the backward purging approach suggests that for both budset and cold hardiness, the first three to five SNPs account for much of the variation, as PVE tended to reach a plateau with only incremental increases with the inclusion of additional SNPs (Figure 4, A and B, respectively). In contrast, randomly selected SNPs collectively had much less explanatory power (less than 5% in most cases) than the optimized models (Figure 4), a result consistent regardless of the number of SNPs from 2 to 20 included in the model.

### Importance values for individual SNPs

Adjusting for population structure did not change the identity of the top two SNPs for budset, whereas for cold injury the top SNP before the adjustment decreased to fourth most important following population adjustment (data not shown). For budset, seven of the top 10 SNPs remained within the top 10 after adjustment for population structure, and all of the top 10 SNPs after adjustment were among the top 15 SNPs in the unadjusted model. For cold hardiness, eight of the top 10 SNPs were in common between the adjusted and unadjusted models, although the ranking changed in a few cases. The 10th most important SNP in the population adjusted model increased in importance from position 24 in the unadjusted model. Of the 339 predictor SNPs, 114 had negative importance values for the budset model (Figure 3A) and 105 had negative values for the cold hardiness model (Figure 3B).

**Figure 5:**
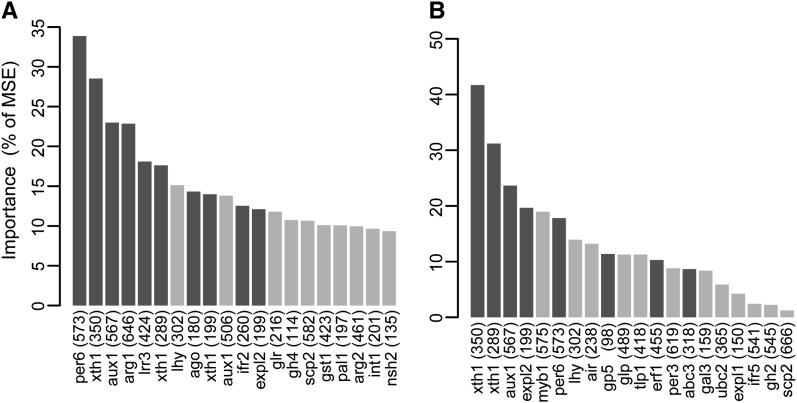
Importance values for top 20 SNPs for budset (A) and cold hardiness (B) identified by the initial run of RF that included all 339 SNPs. SNPs highlighted in dark gray had significant main effects when the mixed model (*after* Holliday *et al.* 2010) was used, and those highlighted in light gray were only significant using RF.

Although RF and the mixed linear model (MLM), previously used by Holliday *et al.* (2010) to identify genotype-phenotype associations, assessed the explanatory power of our SNP markers in different ways, the concordance between the two methods was high for the SNPs with strongest associations with phenotype. This concordance broke down, however, beyond the top 10 SNPs. For budset timing, after adjustment for population structure, eight of the top 10 SNPs identified using RF were also significant using the MLM, whereas only two of the next 10 were in concordance with the MLM (Figure 5A). For cold hardiness, six of the top 10 SNPs and two of the next 10 from the RF analysis were also significant using the MLM (Figure 5B).

### Tests for epistasis

**Figure 6:**
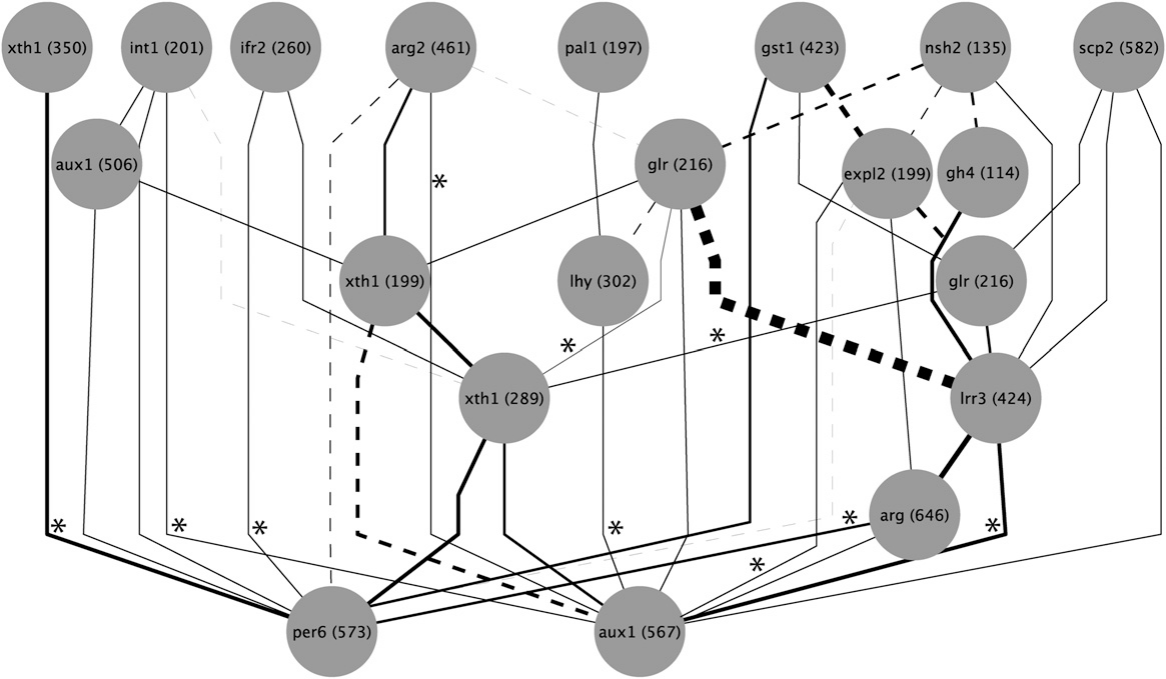
Cytoscape interaction network showing significant (Q < 0.05) pairwise epistatic interactions for budset among the top 20 SNPs measured by their importance values. Line width indicates the relative strength of the interaction; dashed lines indicate negative (antagonistic) epistasis, and solid lines indicate positive (synergistic) epistasis. Asterisks indicate significant genotypic linkage disequilibrium (Q < 0.05) between interacting SNPs.

**Figure 7:**
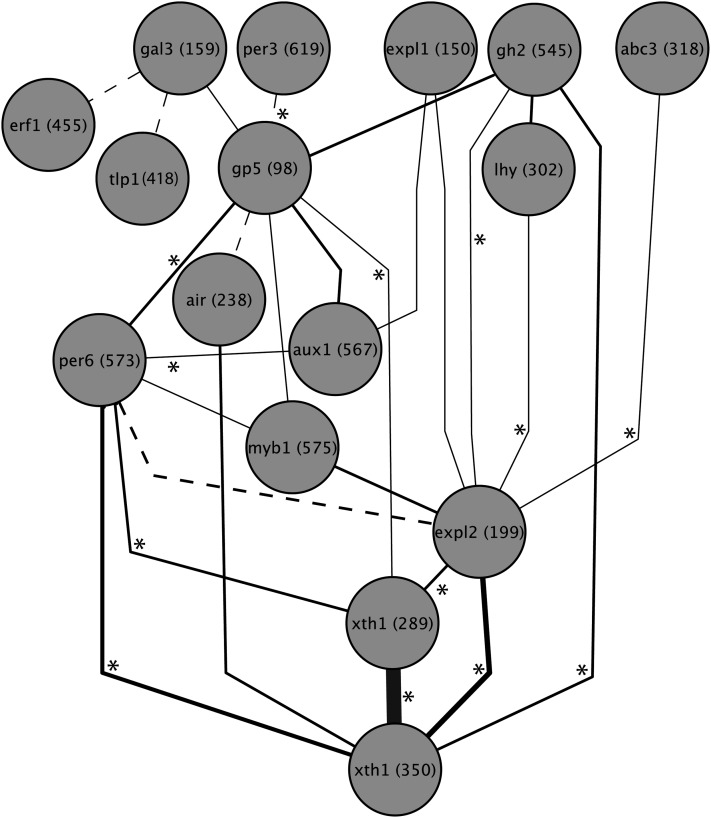
Cytoscape interaction network showing significant (Q < 0.05) pairwise epistatic interactions for cold hardiness among the top 20 SNPs measured by their importance values. Line width indicates the relative strength of the interaction; dashed lines indicate negative (antagonistic) epistasis, and solid lines indicate positive (synergistic) epistasis. Asterisks indicate significant genotypic linkage disequilibrium (Q < 0.05) between interacting SNPs.

By sequentially removing SNPs from the model and recalculating importance values for those that remained, we were able to quantify cases of both synergistic and antagonistic epistasis. We conducted this analysis using the top 20 SNPs based on importance values from the initial RF analysis with adjustment for population structure and followed by a backward purging approach described previously, and the resulting epistasis networks were plotted using Cytoscape (Figures 6 and 7) (Shannon *et al.* 2003). Numerous statistically significant interactions were identified among the top SNPs for each trait. A larger number of pairwise interactions were found for budset (45 interactions) than for cold hardiness (27 interactions). Interestingly, the SNPs with the highest importance values for both traits also were among the most highly connected in its respective network. For budset, *per6* (573) had eight interactions, whereas for cold hardiness, *xth1* (350) had five interactions. However, some SNPs with relatively small contributions to the model also exhibited high connectivity, including *gp5* (98), which for cold hardiness had eight interactions. Conversely, the second most important SNP for budset had only one interaction.

Finally, we calculated genotypic linkage disequilibrium for each pair of interacting SNPs. For budset, 10 of 45 SNP pairs that exhibited significant epistatic interactions were also in linkage disequilibrium (Q < 0.05), and for cold hardiness, 13 of 27 interacting SNPs were in LD. Most interacting SNPs were found on different genes, and significant LD was therefore not likely due tight physical linkage in most cases, though our markers were not genetically mapped and we therefore do not know the physical proximity of the candidate genes. However, given the size of the spruce genome (∼18 Gb) (Ahuja and Neale 2005), it is likely that most interacting SNPs are separated by large physical distances or reside on different chromosomes.

## Discussion

We used RF to identify subsets of SNPs that provide the greatest power to predict adaptive phenotypes in the conifer Sitka spruce. This integrated approach revealed that only a few SNPs are needed to capture a large portion of the quantitative variation in adaptive traits, a finding is somewhat surprising because the conventional analysis of variance approach usually identifies a large number of SNPs that contribute relatively small amounts of variation to the phenotype (Eckert *et al.* 2009a, 2012; Quesada *et al.* 2010; Wegrzyn *et al.* 2010). A possible explanation for this difference would be the inclusion of interactions in the RF model used in this study. Specifically, with our backward purging approach, higher-order interactions are considered in reducing the number of SNPs in the model, and direct tests for epistasis showed that it is rampant among these SNPs. The role of interactions in predicting phenotypes has increasingly been recognized (Malmberg *et al.* 2005; von Korff *et al.* 2010; Pavlicev *et al.* 2011) although there are a paucity of data in plants. One study in Arabidopsis showed that the magnitude of epistatic effects on several fitness related traits was roughly double that of the effects of the additive QTL (Malmberg *et al.* 2005). Our results advance an empirical understanding of how populations may adapt to local climate across many loci and should facilitate the screening of climatically relevant adaptive variation in natural, breeding and deployment populations, and to developing species-specific genetic resource management and conservation strategies.

### A small number of SNPs are sufficient to explain much of the variation in adaptive traits in Sitka spruce

Many temperate and boreal plant species have expanded from glacial refugia to occupy wide latitudinal ranges in a relatively short period of time, on the order of 10−15,000 years. Such expansions necessitate ongoing adaptation to novel climates, which for annual plants is not difficult to envision due to their short generation time. However, long-lived tree species present somewhat of an adaptive paradox in that they have a delay until reproductive maturity resulting in a generation time on the order of 20 to 50 years or longer, depending on ecological disturbance or harvesting rotations (Petit and Hampe 2006). This presents two impediments to local adaptation, namely, a lower effective mutation rate and fewer opportunities for allele frequency adjustments. How then have long-lived tree species been able to expand their ranges so rapidly while simultaneously tracking relative phenotypic optima so closely? Our results suggest that although the average effect of individual SNPs on trait variance is small, the genetic architecture of these traits is clearly finite. Of course, this study only accounts for a small fraction of the segregating variation present in the genome of spruce, and future work that capitalizes on next generation sequencing technologies will no doubt improve on the model we developed here.

It should be noted that the variation we explain with this sample, as is the case in many genotype−phenotype association studies of natural tree species, comprises both within and among-population components. Hence, the relevant measure of quantitative genetic variance with which to compare our model is Q_ST_ (the proportion of phenotypic variation attributable to differentiation among populations), which in Sitka spruce for the same populations was reported to be ∼0.89 for both cold hardiness and budset (Mimura and Aitken 2007). Our RF model therefore explains on the order of a third to a half of the variation present in the studied collection of populations. Adjusting for population structure reduced the explanatory power of the SNPs. Although it was necessary to make this adjustment to avoid false positives, Sitka spruce has a very wide latitudinal range but is essentially restricted to coastal habitat, and population structure in this species covaries with selective pressures related to climate. As such, removing the effect of population structure inevitably removes some of the SNP effects of interest, and hence reduces explanatory power (Myles *et al.* 2009). Taken together, our results would seem to support modern refinements of Fisher’s geometric model of adaptation, which emphasizes the importance of a few mutations of moderate effect and many mutations of small effect (Orr 1998, 1999). We have previously shown that SNPs with phenotypic associations in this study segregate in most populations throughout the range of Sitka spruce, including populations well south of the maximum extent of glaciation, and they are therefore likely to be evolutionarily old (Holliday *et al.* 2010). Adaptation to anthropogenic climate change in this species may therefore proceed largely from standing variants, and our results suggest this adaptation may involve fewer loci than is often assumed by the infinitesimal model underlying classical quantitative genetics (Barrett and Schluter 2008). If this is the case, allele frequency adjustments within the loci described here, coupled with realistic migration rates and epistasis, may provide the means for tree populations to track the rapidly changing phenotypic optimums imposed by climate change.

### Epistasis shapes adaptive traits in Sitka spruce

Phenotypes are the product of both genotype and environment according to the central dogma of quantitative genetics, and it is the goal of association genetics to localize genotypic effects while holding environment constant, as in a common garden. These genotypic effects can be further broken down into additive, dominance, and epistatic components. The first two effects can be easily tested in a conventional association analysis, but the computational burden of testing all possible pairwise and higher-order interactions limits our ability to determine the relative contribution of SNP-SNP interactions to complex trait variation. We took a novel three-stage approach to reduce the dimensionality of the data and directly test for epistasis. First, we built a model that took all 339 SNPs as possible predictors. Second, we paired this model down to include only the 20 SNPs with the highest importance values through a backward purging approach. Finally, we iteratively removed individual SNPs and recalculated the importance values of those that remained. This allowed us to build an interaction network describing both the strength and direction of pairwise interactions between adaptive SNPs. The principle behind this approach is that RF assigns an importance value to each SNP based on its contribution to the model either through its main or interactive effect, or both. Therefore, when the model is paired down to a smaller number of SNPs through the backward purging approach, the SNPs remained in the model represent the most important SNPs contributing to the phenotype. If a SNP interacts with other SNPs, the removal of this SNP will affect the importance values of the interacting SNPs. If the importance value of an interacting SNP is reduced, it suggests a positive interaction. Otherwise, the interaction is negative. However, because the magnitude of the interaction is determined based on the change in the importance values rather than the effect on the phenotype as estimated with traditional methods, these interactions only represent the relative importance of the epistasis.

Somewhat surprisingly, all 20 of the most important SNPs for budset and 16 of 20 for cold injury exhibited at least one interaction. This result might be attributable to the backward purging approach we applied because it considered the effect of all levels of interactions for a SNP being removed from the model, which is different from other approaches reported (Jiang *et al.* 2009; Chen *et al.* 2011). SNPs with the highest importance values (and which were significant in the mixed model association tests) for each trait tended to have more interactions than those with lower importance values. One strategy that has been used in the search for epistasis is to specify tests for interactions only among those SNPs with significant univariate associations. Our results suggest that this approach is likely to capture some of the genetic interactions relevant to complex trait variation, but would miss many interactions involving SNPs with no significant main effect. Although we only dealt with the top 20 SNPs from the RF analysis in our tests for epistasis, this is an arbitrary cutoff that could be relaxed to achieve a more comprehensive view of the interaction network governing complex traits.

### Linkage disequilibrium between interacting loci

Epistasis is expected to produce stable LD between interacting loci (Feldman *et al.* 1980; Otto and Feldman 1997). Although LD may also be generated by demographic phenomena such as migration, genetic drift, and single-locus selection (Wade *et al.* 2001), background levels of LD are low in most widespread tree species (Krutovsky and Neale 2005; Gonzalez-Martinez *et al.* 2006a; Eckert *et al.* 2009b). As such, LD between putatively interacting SNPs, particularly for intergenic interactions, provides additional evidence of *bona fide* epistasis. We therefore sought to determine whether putatively interacting SNPs in our study were also in linkage disequilibrium with one another. After correcting for multiple testing, we found that many, although not all, interacting SNPs exhibited LD. Interestingly, all but one of the interacting SNP pairs that also exhibited LD were positive interactions. This result reinforces the finding that negative epistasis promotes recombination, which in turn breaks down LD (Otto and Feldman 1997).

Although most SNPs in this study represented individual genetic loci presumably separated by some physical distance, and likely on different chromosomes in many cases (though we have no genetic map with which to test this assumption), there were a few cases of adjacent SNPs from the same gene among the top 20 in terms of importance values. One of the strongest interactions was for cold hardiness, between two SNPs separated by only 61bp in the *xth1* gene (SNPs 289 and 350). Each of these SNPs, as well as two others within *xth1*, were significantly associated with both budset and cold hardiness and explained between 3% and 5% of the variation in these traits (Holliday *et al.* 2010). Given the close physical proximity of these SNPs, we initially considered the possibility that LD was driving the associations, leading to a high PVE for all the SNPs adjacent to the quantitative trait nucleotide. However, given the strong epistasis for the two SNPs described above, as well as between SNPs 199 and 289 for budset, it appears more likely that each of these SNPs shape the phenotypic traits, alone and in combination. This result is not without precedent: previous studies have identified intragenic epistasis (da Silva *et al.* 2010), and in one case, conformational changes in the protein products were directly linked to epistasis between allelic variants within the relevant gene (Ortlund *et al.* 2007).

This study represents a significant step toward the goal of a comprehensive description of the genomic basis of local adaptation in widely distributed plant species in general, and conifer trees in particular. We conclude that dimensionality reduction using RF, coupled with iterative tests for pairwise interactions, is an efficient and fairly comprehensive approach to uncover SNPs and their interactions that shape adaptive traits, and this approach should be broadly applicable to other systems. The identification of a suite of markers that collectively explain a substantial fraction of the phenotypic variation in budset and cold hardiness in Sitka spruce provides an important resource for the conservation of genetic resources present in wild populations of spruce species, and prediction of ability to adapt to new climates. Future extensions of the RF approach may include the search for interpopulation allelic covariance that some theoretical and empirical work suggests may be responsible for high population differentiation in adaptive traits found in many tree species (Latta 1998; Le Corre and Kremer 2003; Ma *et al.* 2010; Kremer and Le Corre 2011; Le Corre and Kremer 2012). In trees, high Q_ST_ is often accompanied by low F_ST_ (*i.e.,* low genetic differentiation among populations), a result of high pollen-mediated gene flow, and this apparent paradox has yet to be reconciled by so-called F_ST_-outlier approaches that search for markers with unusually high levels of differentiation relative to the neutral distribution. Kremer and Le Corre (2011) show that in such situations, adaptation is likely the result of covariance between alleles at many loci, leading to strong clines in quantitative traits where clines in the underlying adaptive SNPs are weak and indistinguishable from the neutral distribution of F_ST_. Identifying allelic covariance among many possible candidate SNPs is a challenge, and the RF approach could be used to pare down the list of possible allelic combinations that lead to advantageous trait values for different populations. Such an analysis would likely be most successful if relative fitness were used as the response variable rather than the adaptive phenotypes themselves, since the optimal trait value for a given environment would not be known.

## Supplementary Material

Supporting Information

## References

[bib1] Ahuja M. R. Neale D. B., 2005 Evolution of genome size in conifers. Silvae Genet. 54: 126–137

[bib2] Aitken S. N. Yeaman S. Holliday J. A. Wang T. Curtis-McLane S., 2008 Adaptation, migration or extirpation: climate change outcomes for tree populations. Evolutionary Applications 1: 95–111 10.1111/j.1752-4571.2007.00013.x PMC3352395 25567494

[bib3] Barrett R. D. H. Schluter D., 2008 Adaptation from standing genetic variation. Trends Ecol. Evol. 23: 38–44 18006185 10.1016/j.tree.2007.09.008

[bib4] Breiman L., 2001 Random forests. Mach. Learn. 45: 5–32

[bib5] Bureau A. Dupuis J. Falls K. Lunetta K. L. Hayward B., 2005 Identifying SNPs predictive of phenotype using random forests. Genet. Epidemiol. 28: 171–182 15593090 10.1002/gepi.20041

[bib6] Chen C. C. M. Schwender H. Keith J. Nunkesser R. Mengersen K., 2011 Methods for identifying SNP interactions: a review on variations of logic regression, Random Forest and Bayesian logistic regression. Trans. Computat. Biol. Bioinformatics 8: 1580–1591 10.1109/TCBB.2011.46 21383421

[bib7] Cordell H. J., 2009 Detecting gene-gene interactions that underlie human diseases. Nat. Rev. Genet. 10: 392–404 19434077 10.1038/nrg2579 PMC2872761

[bib8] da Silva J. Coetzer M. Nedellec R. Pastore C. Mosier D. E., 2010 Fitness epistasis and constraints on adaptation in a human immunodeficiency virus type 1 protein region. Genetics 185: 293–303 20157005 10.1534/genetics.109.112458 PMC2870964

[bib9] Eckert A. J. Bower A. D. Wegrzyn J. L. Pande B. Jermstad K. D., 2009 a Asssociation genetics of coastal Douglas-fir (*Pseudotsuga menziesii* var. *menziesii*, Pinaceae). I. Cold hardiness-related traits. Genetics 182: 1289–1302 19487566 10.1534/genetics.109.102350 PMC2728866

[bib11] Eckert A. J. Wegrzyn J. L. Pande B. Jermstad K. D. Lee J. M., 2009 b Multilocus patterns of nucleotide diversity and divergence reveal positive selection at candidate genes related to cold hardiness in coastal Douglas-fir (*Pseudotsuga menziesii* var. menziesii). Genetics 183: 289–298 19596906 10.1534/genetics.109.103895 PMC2746152

[bib12] Eckert A. J. Wegrzyn J. L. Cumbie W. P. Goldfarb B. Huber D. A., 2012 Association genetics of the loblolly pine (*Pinus taeda*, Pinaceae) metabolome. New Phytol. 193: 890–902 22129444 10.1111/j.1469-8137.2011.03976.x

[bib13] Feldman M. W. Christiansen F. B. Brooks L. D., 1980 Evolution and recombination in a constant environment. Proc. Natl. Acad. Sci. USA 77: 4838–4841 16592864 10.1073/pnas.77.8.4838 PMC349943

[bib14] Goldstein B. A. Hubbard A. E. Cutler A. Barcellos L. F., 2010 An application of Random Forests to a genome-wide association dataset: methodological considerations & new findings. BMC Genet. 11: 49 20546594 10.1186/1471-2156-11-49 PMC2896336

[bib15] Gonzalez-Martinez S. C. Ersoz E. Brown G. R. Wheeler N. C. Neale D. B., 2006 a DNA sequence variation and selection of tag single-nucleotide polymorphisms at candidate genes for drought-stress response in *Pinus taeda* L. Genetics 172: 1915–1926 16387885 10.1534/genetics.105.047126 PMC1456261

[bib16] Gonzalez-Martinez S. C. Krutovsky K. V. Neale D. B., 2006 b Forest tree population genomics and adaptive evolution. New Phytol. 170: 227–238 16608450 10.1111/j.1469-8137.2006.01686.x

[bib17] Gonzalez-Martinez S. C. Wheeler N. C. Ersoz E. Nelson C. D. Neale D. B., 2007 Association genetics in *Pinus taeda* L. I. Wood property traits. Genetics 175: 399–409 17110498 10.1534/genetics.106.061127 PMC1775017

[bib18] Gonzalez-Martinez S. C. Huber D. Ersoz E. Davis J. M. Neale D. B., 2008 Association genetics in *Pinus taeda* L. II. Carbon isotope discrimination. Heredity 101: 19–26 18478029 10.1038/hdy.2008.21

[bib19] Grattapaglia D. Resende M. D. V., 2011 Genomic selection in forest tree breeding. Tree Genet. Genomes 7: 241–255

[bib20] Hahn L. W. Ritchie M. D. Moore J. H., 2003 Multifactor dimensionality reduction software for detecting gene−gene and gene−environment interactions. Bioinformatics 19: 376–382 12584123 10.1093/bioinformatics/btf869

[bib21] Hamann A. Wang T. L., 2006 Potential effects of climate change on ecosystem and tree species distribution in British Columbia. Ecology 87: 2773–2786 17168022 10.1890/0012-9658(2006)87[2773:peocco]2.0.co;2

[bib22] Hannerz M. Aitken S. N. King J. N. Budge S., 1999 Effects of genetic selection for growth on frost hardiness in western hemlock. Can. J. Forest Res. 29: 509–516

[bib23] Holliday J. A. Ritland K. Aitken S. N., 2010 Widespread, ecologically relevant genetic markers developed from association mapping of climate-related traits in Sitka spruce (*Picea sitchensis*). New Phytol. 188: 501–514 20663060 10.1111/j.1469-8137.2010.03380.x

[bib24] Ingvarsson P. K. Garcia M. V. Luquez V. Hall D. Jansson S., 2008 Nucleotide polymoirphism and phenotypic associations within and around the phytochrome B2 locus in European aspen (*Populus tremula*, Salicaceae). Genetics 178: 2217–2226 18245834 10.1534/genetics.107.082354 PMC2323810

[bib25] Jannink J. L. Lorenz A. J. Iwata H., 2010 Genomic selection in plant breeding: from theory to practice. Brief.Funct. Genomics 9: 166–177 20156985 10.1093/bfgp/elq001

[bib26] Jiang R. Tang W. W. Wu X. B. Fu W. H., 2009 A random forest approach to the detection of epistatic interactions in case-control studies. BMC Bioinformatics 10(Suppl. 1)**:** S65 19208169 10.1186/1471-2105-10-S1-S65 PMC2648748

[bib27] Kremer A. Le Corre V., 2011 Decoupling of differentiation between traits and their underlying genes in response to divergent selection. Heredity 108: 375–385 21915150 10.1038/hdy.2011.81 PMC3313045

[bib28] Krutovsky K. V. Neale D. B., 2005 Nucleotide diversity and linkage disequilibrium in cold-hardiness- and wood quality-related candidate genes in Douglas fir. Genetics 171: 2029–2041 16157674 10.1534/genetics.105.044420 PMC1456123

[bib29] Latta R. G., 1998 Differentiation of allelic frequencies at quantitative trait loci affecting locally adaptive traits. Am. Nat. 151: 283–292 18811359 10.1086/286119

[bib30] Le Corre V. Kremer A., 2003 Genetic variability at neutral markers, quantitative trait loci and trait in a subdivided population under selection. Genetics 164: 1205–1219 12871925 10.1093/genetics/164.3.1205 PMC1462636

[bib31] Le Corre V. Kremer A., 2012 The genetic differentiation at quantitative trait loci under local adaptation. Mol. Ecol. 21: 1548–1566 22332667 10.1111/j.1365-294X.2012.05479.x

[bib32] Liaw A. Wiener M., 2002 Classification and regression by randomForest. R News 2: 18–22

[bib33] Lunetta K. L. Hayward L. B. Segal J. Van Eerdewegh P., 2004 Screening large-scale association study data: exploiting interactions using random forests. BMC Genet. 5: 32 15588316 10.1186/1471-2156-5-32 PMC545646

[bib34] Lunn D. J. Whittaker J. C. Best N., 2006 A Bayesian toolkit for genetic association studies. Genet. Epidemiol. 30: 231–247 16544290 10.1002/gepi.20140

[bib35] Ma X.-F. Hall D. St Onge K. R. Jansson S. Ingvarsson P. K., 2010 Genetic differentiation, clinal variation and phenotypic associations with growth cessation across the populus tremula photoperiodic Pathway. Genetics 186: 1033–1044 20805554 10.1534/genetics.110.120873 PMC2972289

[bib36] Malcolm J. R. Markham A. Neilson R. P. Garaci M., 2002 Estimated migration rates under scenarios of global climate change. J. Biogeogr. 29: 835–849

[bib37] Malmberg R. L. Held S. Waits A. Mauricio R., 2005 Epistasis for fitness-related quantitative traits in Arabidopsis thaliana grown in the field and in the greenhouse. Genetics 171: 2013–2027 16157670 10.1534/genetics.105.046078 PMC1456117

[bib38] McLachlan J. S. Clark J. S. Manos P. S., 2005 Molecular indicators of tree migration capacity under rapid climate change. Ecology 86: 2088–2098

[bib39] Meuwissen T. H. E. Hayes B. J. Goddard M. E., 2001 Prediction of total genetic value using genome-wide dense marker maps. Genetics 157: 1819–1829 11290733 10.1093/genetics/157.4.1819 PMC1461589

[bib40] Mimura M. Aitken S. N., 2007 Adaptive gradients and isolation-by-distance with postglacial migration in *Picea sitchensis*. Heredity 99: 224–232 17487214 10.1038/sj.hdy.6800987

[bib41] Moore J. H. Gilbert J. C. Tsai C. T. Chiang F. T. Holden T., 2006 A flexible computational framework for detecting, characterizing, and interpreting statistical patterns of epistasis in genetic studies of human disease susceptibility. J. Theor. Biol. 241: 252–261 16457852 10.1016/j.jtbi.2005.11.036

[bib42] Myles S. Peiffer J. Brown P. J. Ersoz E. S. Zhang Z. W., 2009 Association mapping: critical considerations shift from genotyping to experimental design. Plant Cell 21: 2194–2202 19654263 10.1105/tpc.109.068437 PMC2751942

[bib43] Neale D. B. Savolainen O., 2004 Association genetics of complex traits in conifers. Trends Plant Sci. 9: 325–330 15231277 10.1016/j.tplants.2004.05.006

[bib44] Nemri A. Atwell S. Tarone A. M. Huang Y. S. Zhao K., 2010 Genome-wide survey of Arabidopsis natural variation in downy mildew resistance using combined association and linkage mapping. Proc. Natl. Acad. Sci. USA 107: 10302–10307 20479233 10.1073/pnas.0913160107 PMC2890483

[bib45] Orr H. A., 1998 The population genetics of adaptation: the distribution of factors fixed during adaptive evolution. Evolution 52: 935–949 10.1111/j.1558-5646.1998.tb01823.x 28565213

[bib46] Orr H. A., 1999 The evolutionary genetics of adaptation: a simulation study. Genet. Res. 74: 207–214 10689798 10.1017/s0016672399004164

[bib47] Ortlund E. A. Bridgham J. T. Redinbo M. R. Thornton J. W., 2007 Crystal structure of an ancient protein: evolution by conformational epistasis. Science 317: 1544–1548 17702911 10.1126/science.1142819 PMC2519897

[bib48] Otto S. P. Feldman M. W., 1997 Deleterious mutations, variable epistatic interactions, and the evolution of recombination. Theor. Popul. Biol. 51: 134–147 9169238 10.1006/tpbi.1997.1301

[bib49] Pavlicev M. Norgard E. A. Fawcett G. L. Cheverud J. M., 2011 Evolution of pleiotropy: epistatic interaction pattern supports a mechanistic model underlying variation in genotype-phenotype map. J. Exp. Zoolog. B Mol. Dev. Evol. 316B: 371–385 10.1002/jez.b.21410 PMC3112255 21462316

[bib50] Petit R. J. Hampe A., 2006 Some evolutionary consequences of being a tree. Annu. Rev. Ecol. Evol. Syst. 37: 187–214

[bib51] Phillips P. C., 2008 Epistasis—the essential role of gene interactions in the structure and evolution of genetic systems. Nat. Rev. Genet. 9: 855–867 18852697 10.1038/nrg2452 PMC2689140

[bib52] Pritchard J. K. Stephens M. Donnelly P., 2000 Inference of population structure using multilocus genotype data. Genetics 155: 945–959 10835412 10.1093/genetics/155.2.945 PMC1461096

[bib53] Quesada T. Gopal V. Cumbie W. P. Eckert A. J. Wegrzyn J. L., 2010 Association mapping of quantitative disease resistance in a natural population of loblolly pine (*Pinus taeda* L.). Genetics 186: 677–686 20628037 10.1534/genetics.110.117549 PMC2954465

[bib54] Resende M. F. R. Munoz P. Acosta J. J. Peter G. F. Davis J. M., 2012 Accelerating the domestication of trees using genomic selection: accuracy of prediction models across ages and environments. New Phytol. 193: 617–624 21973055 10.1111/j.1469-8137.2011.03895.x

[bib55] Rousset F., 2008 GENEPOP’007: a complete re-implementation of the GENEPOP software for Windows and Linux. Molecular Ecology Resources 8: 103–106 21585727 10.1111/j.1471-8286.2007.01931.x

[bib56] Savolainen O. Pyhajarvi T. Knurr T., 2007 Gene flow and local adaptation in tees. Annu. Rev. Ecol. Evol. Syst. 38: 595–619

[bib57] Shannon P. Markiel A. Ozier O. Baliga N. S. Wang J. T., 2003 Cytoscape: a software environment for integrated models of biomolecular interaction networks. Genome Res. 13: 2498–2504 14597658 10.1101/gr.1239303 PMC403769

[bib58] Storey J. D. Tibshirani R., 2003 Statistical significance for genomewide studies. Proc. Natl. Acad. Sci. USA 100: 9440–9445 12883005 10.1073/pnas.1530509100 PMC170937

[bib59] von Korff M. Leon J. Pillen K., 2010 Detection of epistatic interactions between exotic alleles introgressed from wild barley (*H. vulgare ssp. spontaneum*). Theor. Appl. Genet. 121: 1455–1464 20617300 10.1007/s00122-010-1401-y

[bib60] Wade M. J. Winther R. G. Agrawal A. F. Goodnight C. J., 2001 Alternative definitions of epistasis: dependence and interaction. Trends Ecol. Evol. 16: 498–504

[bib61] Wegrzyn J. L. Eckert A. J. Choi M. Lee J. M. Stanton B. J., 2010 Association genetics of traits controlling lignin and cellulose biosynthesis in black cottonwood (*Populus trichocarpa*, Salicaceae) secondary xylem. New Phytol. 188: 515–532 20831625 10.1111/j.1469-8137.2010.03415.x

[bib62] Yu J. M. Pressoir G. Briggs W. H. Bi I. V. Yamasaki M., 2006 A unified mixed-model method for association mapping that accounts for multiple levels of relatedness. Nat. Genet. 38: 203–208 16380716 10.1038/ng1702

[bib63] Zhang Y. Liu J. S., 2007 Bayesian inference of epistatic interactions in case-control studies. Nat. Genet. 39: 1167–1173 17721534 10.1038/ng2110

