# Supplementary material for: Predicting Adaptive Phenotypes From Multilocus Genotypes in Sitka Spruce (Picea sitchensis) Using Random Forest
Source: G3 (Bethesda). 2012 Sep 1;2(9):1085–93. doi: 10.1534/g3.112.002733 (PMC3429923; doi:10.1534/g3.112.002733)
Supplement: Supporting Information [file supp_2_9_1085__index.html]

Supporting Information 

# Predicting Adaptive Phenotypes From Multilocus Genotypes in Sitka Spruce (*Picea sitchensis*) Using Random Forest

## Supporting Information for Holliday, Wang, and Aitken, 2012

**Files in this Data Supplement:**

- File S1 - R script for Random Forest analysis with SNPs data (PDF, 57 KB)
- Table S1 - Origins of study populations and cluster assignments from structure software (.xlsx, 49 KB)
- Table S2 - Candidate genes employed in this study (.xlsx, 60 KB)
- Table S3 - Phenotype and genotype data for budset (.xlsx, 480 KB)
