# Supplementary material for: Predicting Adaptive Phenotypes From Multilocus Genotypes in Sitka Spruce (Picea sitchensis) Using Random Forest
Source: G3 (Bethesda). 2012 Sep 1;2(9):1085–93. doi: 10.1534/g3.112.002733 (PMC3429923; doi:10.1534/g3.112.002733)
Supplement: Supporting Information [file supp_2.9.1085_FileS1.pdf]

File S1

R script for Random Forest analysis with SNPs data

Random Forest analysis with all SNPs

```
## Initiation
library(randomForest)
setwd("Path_to_working_directory")

# #Import dataset to R
# Both phenotype and SNPs data (xy0)
xy0<-read.table("your_file_name")

#Create a dataset for SNPs (x0)
x0<-select_columns_of_SNPs

#create a dataset for phenotype (y)
y<-select_columns_with_phenotypes

## Random Forest (RF) analysis  with all SNPs
myrf<-randomForest(x0, y, ntree=1500, importance=TRUE)

#Show results and save the importance values
print(myrf)
write.csv(round(importance(myrf),2), "Importance_values_all.csv")

#Independent predictions using Out-Of-Bag data samples
#Combine predictions and observation into a single dataset
p1<- predict(myrf) #OOB predictions
yp<-cbind(p1,y)
write.csv(yp, "yp.csv")
```

Random Forest backward purging process with 50 most important SNPs

```
# 1) first to included 50 SNPs in x50;
# 2) remove the least important SNP based on their importance values;
# 3) repeated 3 runs to determine the least important SNP based on the times having the smallest importance value;
# 4) repeat above to remove SNPs one by one

#50 SNPs (the base dataset, using budset data as an example)
x50<- create_matrix_of_50_most_important_SNPs

# the dataset for analysis with least important SNPs removed one by one
xr<- x50[,c("209_523_S","113_189_S")]

        imp1<-NULL
        imp2<-NULL
        ntree=1500
        rf1<-randomForest(xr, y, ntree=ntree,importance=TRUE); print(rf1)
        rf2<-randomForest(xr, y, ntree=ntree,importance=TRUE); print(rf2)
        rf3<-randomForest(xr, y, ntree=ntree,importance=TRUE); print(rf3)

        lsv=10
        imp1<-(importance(rf1, type=1))
        mn<-min(imp1)
```

```
        imp2$impv<-imp1[imp1<=mn+lsv+1,] #change the constant to get ...
        imp2
        imp1<-(importance(rf2, type=1))
        mn<-min(imp1)
        imp2$impv<-imp1[imp1<=mn+lsv+1,] #change the constant to get ...
        imp2
        imp1<-(importance(rf3, type=1))
        mn<-min(imp1)
        imp2$impv<-imp1[imp1<=mn+lsv+1,] #change the constant to get ...
        imp2
 dim(xr)
```

Random Forest analysis with the top 20 SNPs

```
# The 20 top important SNPs (for budset as an example)
x20<-create_matrix_of_top_20_SNPs

#Independent predictions with 20 SNPs
rf20<-randomForest(x20, y, ntree=2000,importance=TRUE)
p1<- predict(rf20)
yp<-cbind(p1,y)
write.csv(yp, "yp_bs.csv")
```

Random Forest analysis of interactions among the top 20 SNPs

```
#Backward interactions
# 5 runs for x20 to get importance values
nt=1500
for (i in 1:10) {
        rf20<-randomForest(x20, y, ntree=nt,importance=TRUE)
        imp1<-round(importance(rf20, type=1),2)
        if (i<2) { impx<-imp1 }
        if (i>1) {impx<-cbind(impx,imp1) }
}
write.csv(impx,"interact/bs_imp20n_5runs.csv")

# remove one SNP at a time to see changes in the importance values of the remaining 19 SNPs
# run1
for (j in 1:20) {
        x3 <- x20[,-j]
        rf19<-randomForest(x3, y, ntree=nt,importance=TRUE)
        imp1<-round(importance(rf19, type=1),2)
        if (j<2) { impx<-imp1 }
        if (j>1) {impx<-cbind(impx,imp1) }
}
write.csv(impx,"interact/imp19_run1.csv")

# run2
for (j in 1:20) {
        x3 <- x20[,-j]
        rf19<-randomForest(x3, y, ntree=nt,importance=TRUE)
        imp1<-round(importance(rf19, type=1),2)
        if (j<2) { impx<-imp1 }
        if (j>1) {impx<-cbind(impx,imp1) }
}
write.csv(impx,"interact/imp19_run2.csv")
```

```
# run3
for (j in 1:20) {
		x3 <- x20[,-j]
		rf19<-randomForest(x3, y, ntree=nt,importance=TRUE)
		imp1<-round(importance(rf19, type=1),2)
		if (j<2) { impx<-imp1 }
		if (j>1) {impx<-cbind(impx,imp1) }
}
write.csv(impx,"interact/imp19_run3.csv")

# run4
for (j in 1:20) {
		x3 <- x20[,-j]
		rf19<-randomForest(x3, y, ntree=nt,importance=TRUE)
		imp1<-round(importance(rf19, type=1),2)
		if (j<2) { impx<-imp1 }
		if (j>1) {impx<-cbind(impx,imp1) }
}
write.csv(impx,"interact/imp19_run4.csv")

# run5
for (j in 1:20) {
		x3 <- x20[,-j]
		rf19<-randomForest(x3, y, ntree=nt,importance=TRUE)
		imp1<-round(importance(rf19, type=1),2)
		if (j<2) { impx<-imp1 }
		if (j>1) {impx<-cbind(impx,imp1) }
}
write.csv(impx,"interact/imp19_run5.csv")
```
